# Polarity-inverted lateral overgrowth and selective wet-etching and regrowth (PILOSWER) of GaN

**DOI:** 10.1038/s41598-018-22424-4

**Published:** 2018-03-07

**Authors:** Dongsoo Jang, Miyeon Jue, Donghoi Kim, Hwa Seob Kim, Hyunkyu Lee, Chinkyo Kim

**Affiliations:** Department of Physics and Research Institute for Basic Sciences, Kyung Hee University, 26 Kyungheedae-ro, Dongdaemun-gu, Seoul 02447 Korea

## Abstract

On an SiO_2_-patterned *c*-plane sapphire substrate, GaN domains were grown with their polarity controlled in accordance with the pattern. While N-polar GaN was grown on hexagonally arranged circular openings, Ga-polar GaN was laterally overgrown on mask regions due to polarity inversion occurring at the boundary of the circular openings. After etching of N-polar GaN on the circular openings by H_3_PO_4_, this template was coated with 40-nm Si by sputtering and was slightly etched by KOH. After slight etching, a thin layer of Si left on the circular openings of sapphire,but not on GaN, was oxidized during thermal annealing and served as a dielectric mask during subsequent regrowth. Thus, the subsequent growth of GaN was made only on the existing Ga-polar GaN domains, not on the circular openings of the sapphire substrate. Transmission electron microscopy analysis revealed no sign of threading dislocations in this film. This approach may help fabricating an unholed and merged GaN film physically attached to but epitaxially separated from the SiO_2_-patterned sapphire.

## Introduction

Due to large mismatch of lattice constants between GaN and sapphire, heteroepitaxial growth of GaN on a sapphire substrate inevitably introduces undesirable threading dislocations (TDs), which deteriorate the performance of GaN/sapphire-based optoelectronic devices. Epitaxial lateral overgrowth (ELOG) was one of the first methods to effectively reduce TDs in heteroepitaxial growth of *c*-GaN^1^. Although this method was effective in reducing TDs in a mask region, a high density of dislocations was still commonly observed in window opening regions unless additional facet control was accompanied during the overgrowth^2^. Similar variation such as pendeo epitaxy^3^ was proposed, and sidewall lateral epitaxial overgrowth^4^ turned out to be effective in reducing dislocations in semipolar or nonpolar GaN.

In fact, the extended-defect-control issue of GaN when heteroepitaxially grown on a foreign substrate originates from difficulty in growing a GaN single crystal boule, so that many attempts have been alternatively made to obtain a free-standing GaN substrate by eliminating a foreign substrate on which GaN was heteroepitaxially grown. Among various approaches, the removal methods of a sapphire substrate such as laser lift off (LLO)^5–7^, void-assisted self-separation (VAS)^8–10^, and chemical lift off (CLO)^11,12^ were extensively studied. Stress and damages caused by thermal shock during LLO were, however, reported to be problematic^13,14^. VAS requires growth of a thick (at least a few hundred microns) GaN for self-separation. On the other hand, CLO, which is free from thermal shock during a separation process, requires a wet-etchable sacrificial layer, which typically resulted in degradation of crystalline quality of GaN grown upon it. It was, however, reported that a relatively good crystalline free-standing GaN layer could be obtained by using lateral overgrowth on a GaN/sapphire template with an SiO_2_-coated tungsten mask, which was found to react with GaN to form voids in such a way that subsequently grown GaN film could be separated by wet chemical etching, but GaN grown on window regions still contained a high density of dislocations^15^. Thus, these approaches to fabricating free standing GaN are still in need of improvement to grow high crystalline (but not necessarily a-few-hundred-micron-thick) as well as easily separable GaN.

A non-centrosymmetric crystal structure of Wurtzite GaN makes N-polar GaN behave very differently from Ga-polar GaN. In terms of chemical reactivity, an N-polar facet is easily etched by KOH or H_3_PO_4_ while a Ga-polar facet is very inert to chemical etchants^16,17^. This chemical reactivity difference allowed polarity-selective chemical etching to be utilized for patterning a polarity-mixed GaN layer^18^. Recently it was reported that the polarity of GaN domains inverted from N polarity to Ga polarity at the boundary of a dielectric mask during ELOG^19,20^. Song *et al*. focused on suppression of an inversion process from N- to Ga-polarity, so that they were able to laterally overgrow a continuous N-polar GaN film^20^. On the other hand, Wang *et al*. enhanced vertical growth of N-polar GaN in window regions while they suppressed the growth rate of Ga-polar GaN around the window openings, so that N-polar GaN columns were successfully grown on the window openings^19^. In those papers, the growth of a singles N-polar GaN structure (either film or micro column) was a main goal. It, however, came to our attention that a polarity-controlled GaN film can be obtained if the polarity inversion during ELOG is properly enhanced. Once the selective growth of Ga-polar GaN on mask regions and N-polar GaN on window regions are completed, N-polar GaN on window regions can be selectively removed by wet etching in such a way that high crystalline Ga-polar GaN domains on the mask regions can be obtained. By selectively regrowing GaN on the existing GaN domains, not on the exposed sapphire surface in the window regions, a thin continuous GaN film, which is epitaxially separated from but physically attached to the SiO_2_-patterned sapphire substrate, can be obtained. This type of a thin GaN film on sapphire can serve as effectively as a free-standing thick substrate because this GaN thin film and the substrate are epitaxially decoupled. Since a thin GaN layer on SiO_2_ is readily separable from the substrate by HF solution, this template can serve as an excellent platform for fabricating a free-standing GaN substrate as well. In this work, we propose and demonstrate polarity-inverted lateral overgrowth and selective wet-etch and regrowth (PILOSWER), which can be adopted for fabricating a GaN film epitaxially separated from but physically attached to an SiO_2_-patterned sapphire substrate. The main feature of our proposed method is that defective GaN, which is grown intentionally with N-polarity on circular openings, can be selectively removed by wet etching in such a way that a threading-dislocation-free *thin* GaN layer can become readily separable from the substrate without a foreign sacrificial layer while the physical attachment of the thin film to the substrate is maintained.

## Results and Discussion

PILOSWER consists of three steps: (1) initial growth including nitridation and lateral overgrowth, (2) selective wet etch and (3) regrowth. For the successful realization of PILOSWER, several factors should be satisfied in all three steps. We describe the roles of these factors one by one in the following paragraphs.

**Figure 1:**
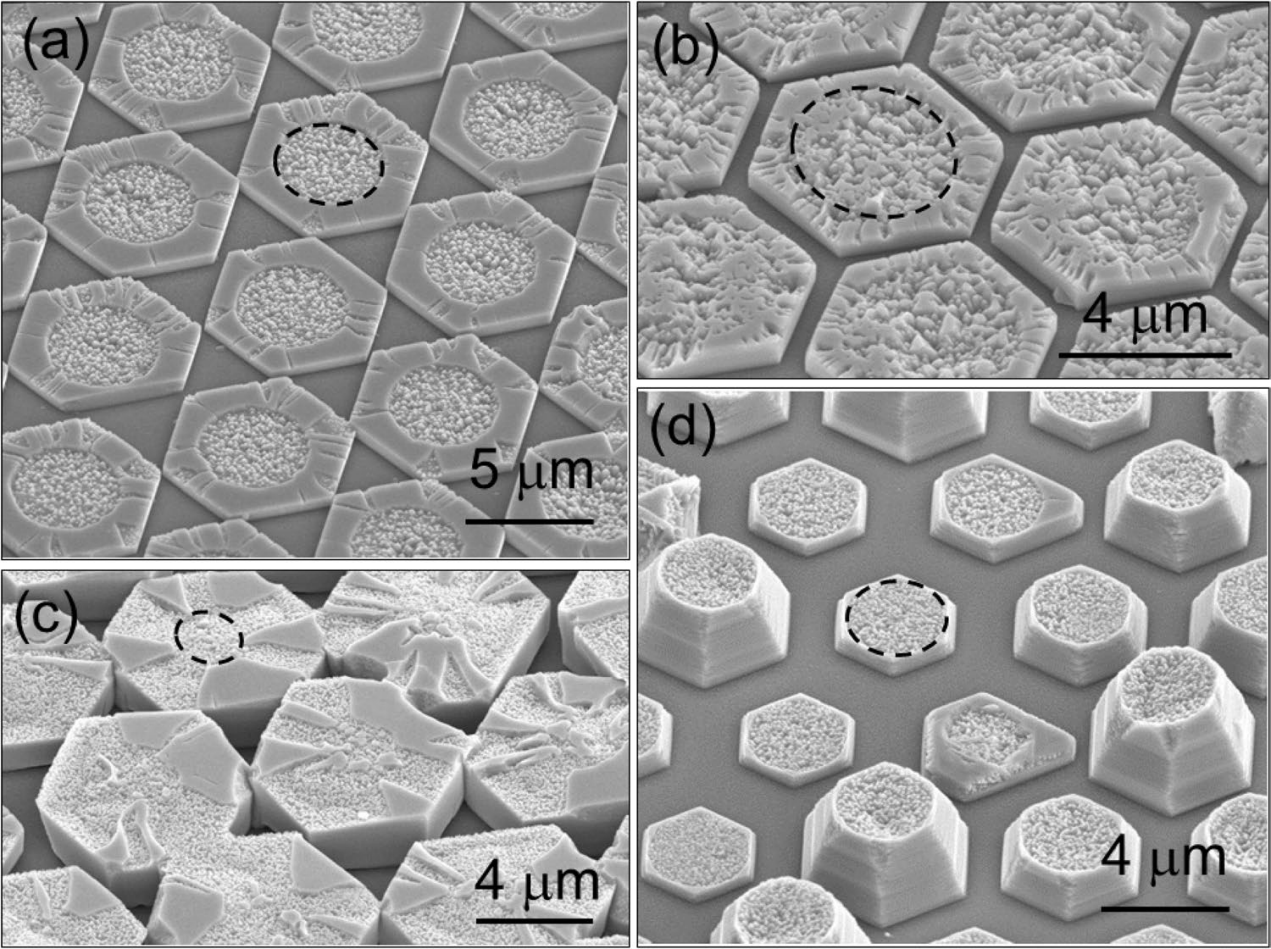
Various samples grown under different growth conditions were etched by KOH for polarity check. Secondary electron images (SEIs) of slightly KOH-etched samples show different surface morphologies as described in the following. (**a**) Nitridation, polarity inversion and uniform growth of individual domains were sufficiently done. (**b**) Submicron pyramidal hillocks were observed to be concentrated inside the circular region, but there were also locally flat top facets inside circular regions. This morphology suggests that Ga- and -N polarity were mixed in the circular region. (**c**) High concentration of submicron hillocks were observed in the overgrown region, which suggests that polarity inversion at the circular boundary occurred only partially. (**d**) The size and height of individual domains were not uniform. Dashed circles mark the boundary of the circular openings. The hexagonal pattern of circular openings in (**a**) and (**c**) is 90°-rotated with respect to the substrate.

In the first step, three factors were found to be important: sufficient nitridation of circular openings on a *c*-plane sapphire substrate, efficient inversion of polarity at the boundary of an SiO_2_ pattern, uniform growth of individual domains. The samples in Fig. 1 show the surface morphology of KOH-etched GaN domains grown in different growth conditions on SiO_2_-patterned *c*-plane sapphire substrates with hexagonally arranged circular openings. When the three factors mentioned above were well satisfied, hexagonally-shaped GaN domains with well developed facets were formed as shown in Fig. 1(a). For this sample, nitridation was carried out for 20 minutes with 4 slm of NH_3_, and GaN was grown at 900 °C with 4 slm of NH_3_ and 10 sccm of HCl flow rates. Individual hexagonally-shaped GaN domains were enclosed by *c*-facet (top surface) and *m*-facets (vertical side walls). The size of the individual GaN domains was larger than that of the circular opening (marked by a black dashed circle), so that the region outside the circle in the individual hexagonal domains was laterally overgrown. This sample reveals that the circular region of individual GaN domains is fully N-polar while the region outside the circle is Ga-polar except for several narrow regions. 100% polarity inversion was rarely observed in our experiments. As mentioned in the introductory part, each domain was found to experience polarity inversion exactly at the boundary of each circular openings.

A case, in which nitridation was not done, is shown in Fig. 1(b). For this sample, nitridation was not carried out, and GaN was grown at 900 °C with 80 sccm of NH_3_ and 10 sccm of HCl flow rates. This surface morphology with pyramidal hillocks and flat regions mixed suggests that the circular regions contained both Ga- and N-polar GaN. Ga-polar GaN in the circular openings could not be wet etched, so this type of sample could not be used for regrowth. Figure 1(c) illustrates a case in which the polarity inversion from N-polarity to Ga-polarity at the circular boundary was not sufficiently done. For this sample, nitridation was carried out for 20 minutes with 4 slm of NH_3_, and GaN was grown at 970 °C with 200 sccm of NH_3_ and 10 sccm of HCl flow rates. In this sample, the height of the hexagonal domains was much larger than that of domains in Fig. 1(a) while the size of the individual domains is comparable with that of domains in Fig. 1(a). This result reveals that N-polarity did not effectively invert to Ga-polarity at the circular boundary if the vertical growth rate was too fast, which had been also pointed out in Song’s paper^20^. On the other hand, there was a case in which the height of individual domains varied as illustrated in Fig. 1(d). For this sample, nitridation was carried out for 10 minutes with 3 slm of NH_3_, and GaN was grown at 900 °C with 3 slm of NH_3_ and 5 sccm of HCl flow rates. In addition to variation of heights, there is one more point which deserve discussion in Fig. 1(d): non-vertical sidewall of each domain. Securing vertical sidewalls is important because the reduction of TDs in ELOG is known to be closely related to whether a sidewall of GaN domains is vertical or tilted^2,21–23^. More discussion regarding the reduction of TDs and the vertical sidewall is given in the latter paragraph describing transmission electron microscopy (TEM) results.

**Figure 2:**
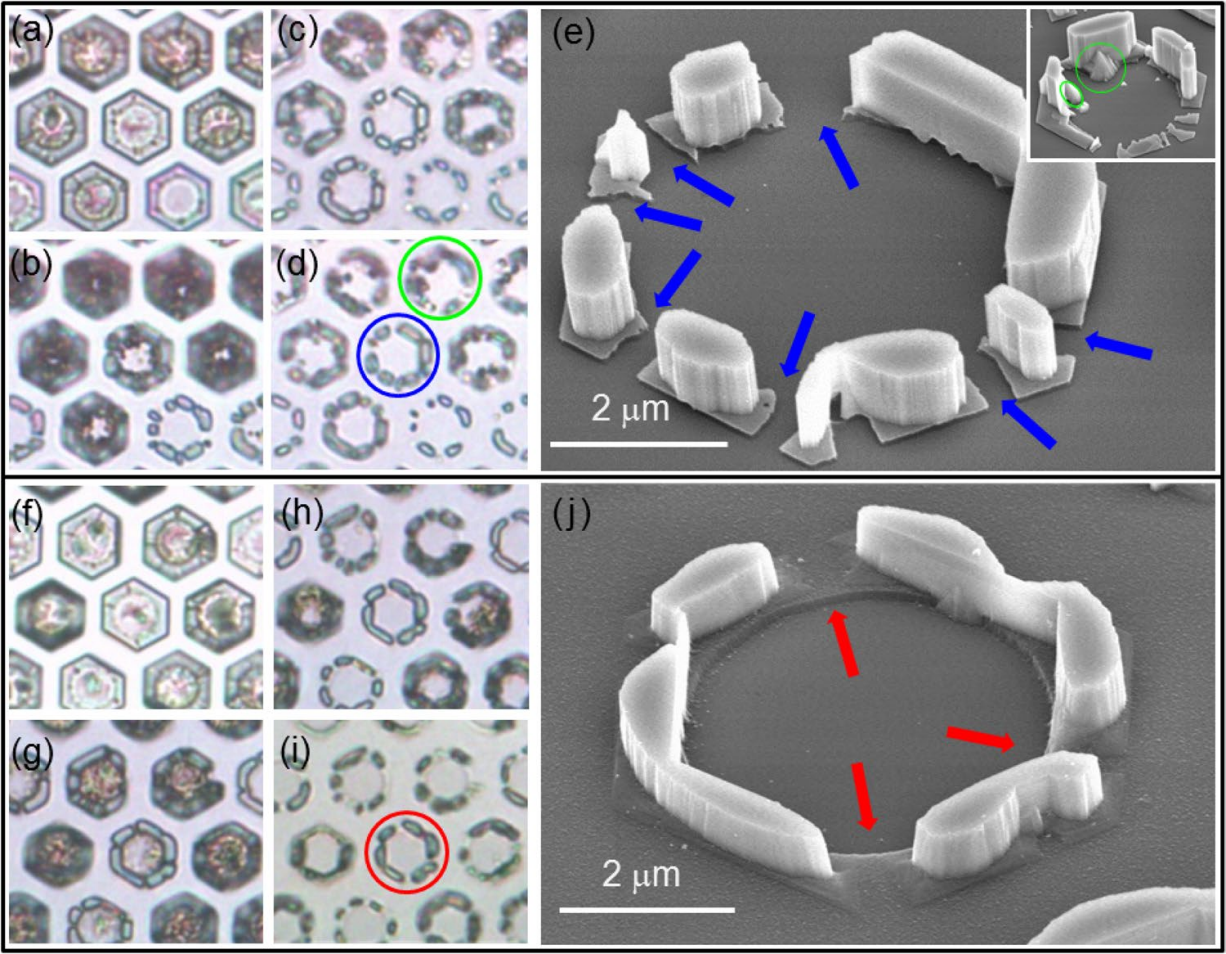
Optical microscopy images of KOH-etched GaN at 50 °C for (**a**) 0 hours (as-grown), (**b**) 2 hours, (**c**) 5 hours, and (**d**) 7 hours. (**e**) SEI of etched GaN marked by a blue circle in (**d**). The regions marked by blue arrows represent where narrow N-polar regions were on the SiO_2_ mask. An inset is an SEI of etched GaN marked by a green circle in (**d**). The dark spots inside a circular opening marked by a green circle in (**d**) turned out to be unetched N-polar GaN, which are marked by green circles in an inset. Optical microscopy images of H_3_PO_4_-etched GaN at 85 °C for (**f**) 0 hours(as-grown), (**g**) 2 hours, (**h**) 5 hours, and (**i**) 7 hours. (**j**) SEI of H_3_PO_4_-etched GaN marked by a red circle in (**i**). The regions marked by red arrows represent where narrow N-polar regions were on the SiO_2_ mask.

In the second step, the effective elimination of N-polar GaN in the circular openings with either minimum or no damage to other parts of the sample is of great importance. Figure 2(a)∼(e) show morphological change of GaN domains, which were etched by KOH solution. The image of as-grown sample (Fig. 2(a)) shows hexagonal-shaped GaN domains grown at each circular opening. After 2-hour etching (Fig. 2(b)), some N-polar regions were completely removed while not fully etched N-polar regions appear dark due to a roughened surface by KOH etching. Note that there were small narrow N-polar regions on SiO_2_ mask because of incomplete polarity inversion in such a way that these N-polar regions were also removed during further KOH etching. After 5-hour etching (Fig. 2(c)), most of N-polar regions were etched out. After 7-hour etching (Fig. 2(d)), only Ga-polar regions survived while N-polar regions almost disappeared. Consequently, Ga-polar GaN seeds for regrowth, which originated from one single hole, were not connected one another. 7-hour KOH etching, however, eliminated most of SiO_2_ mask pattern (except for the region where Ga-polar GaN was located) as well as N-polar GaN as illustrated in Fig. 2(e). In addition, there were unremoved N-polar GaN domains which were still attached to the sapphire substrate even after 7-hour KOH etching (see an inset of Fig. 2(e)). The damage to SiO_2_ pattern by KOH etching could be reduced by decreasing etching duration, but reduced etching duration inevitably increased the volume of unetched N-polar GaN in circular openings. Thus, we looked for alternative etchant, and it turned out that H_3_PO_4_ could effectively remove N-polar GaN without damaging SiO_2_. As shown in Fig. 2(f)∼(j), H_3_PO_4_ etched GaN in a very similar manner, but SiO_2_ was almost not damaged by H_3_PO_4_ etching.

**Figure 3:**
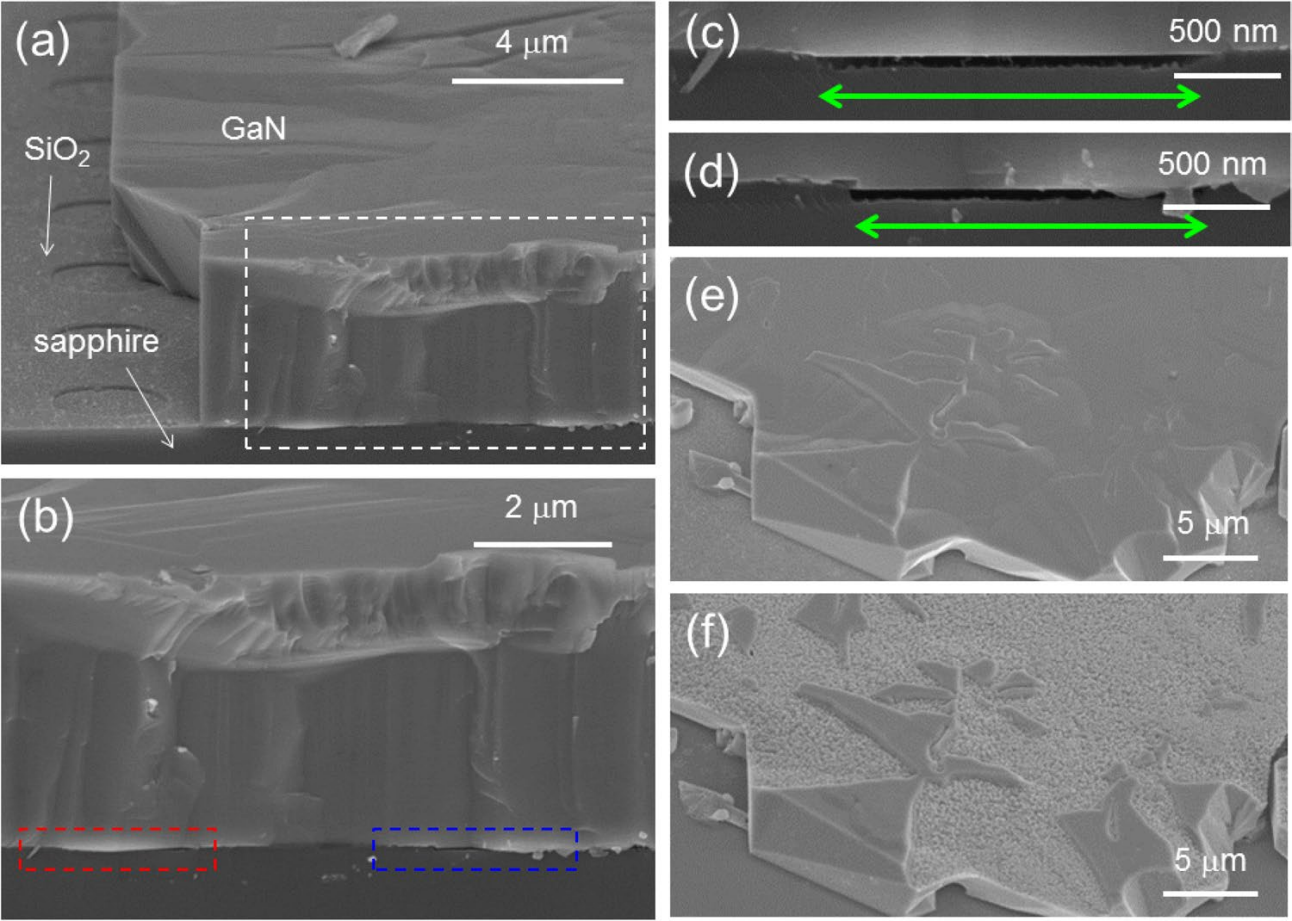
SEIs of GaN after regrowth. (**a**) A tilt view of a regrown GaN film on sapphire, (**b**) a magnified cross-sectional view of the region marked by a dashed white rectangle in (**a**). A magnified cross-sectional view of the region marked by a dashed (**c**) red and (**d**) blue rectangle in (**b**), respectively. Green arrows mark the size of openings. Gaps between regrown GaN and sapphire are clearly seen since no nucleation of GaN was possible on the passivated sapphire surface. SEIs of the surface of another regrown sample (**e**) before and (**f**) after KOH-etching. Surface roughness is clearly associated with mixed polarity.

In order for this holed GaN on SiO_2_/sapphire to be used as an epitaxially separated GaN template, a selective regrowth of GaN should be done, which is the third step for PILOSWER. In this third step, it is crucial to maintain epitaxial separation between GaN and a substrate. For this purpose, GaN should be regrown only on the existing GaN seeds, not on the exposed sapphire substrate. If no special measure is taken regarding regrowth, regrowth of GaN only on the existing seeds may or may not be possible depending on growth conditions and geometrical factors (such as size and depth of openings and separation between openings). On the other hand, if the nucleation sites of the exposed surface of sapphire become unavailable by surface passivation, regrowth of GaN only on the existing GaN seeds is readily doable. It was previously reported that a thin layer of SiO_*x*_ was formed between Al_2_O_3_ and Si under thermal annealing process^24^. So, the deposition and thermal annealing of Si film were adopted in this work for passivating the exposed sapphire surface. 40-nm-thick Si was coated on the H_3_PO_4_-etched GaN/SiO_2_/sapphire template, and this template was etched in 2 M KOH solution at 50 °C for 15 minutes before this template was loaded into the growth reactor for the subsequent regrowth. 15-minute-long KOH etching almost fully removed Si on GaN, but there was a thin layer of Si left on sapphire. There was no explicit step for thermal annealing of Si-coated template, but it was thermally annealed while the template was being heated up inside a growth reactor for regrowth. Then, the subsequent regrowth of GaN after this selective passivation process was carried out on this template. (The detailed analysis of sapphire surface passivation is given in the latter paragraph.) Fig. 3 shows a cross-sectional view of regrown GaN. As illustrated in Fig. 3(c) and (d), there was a gap between regrown GaN and a sapphire substrate. This result suggests that surface passivation worked only for sapphire, not for GaN in such a way that no nucleation of GaN occurred on the sapphire while regrown GaN domains filled up the window region without touching sapphire. As a consequence, the epitaxial separation of GaN from the sapphire substrate was maintained during the regrowth. This regrowth is very similar to pendeo epitaxy, but a great difference between our case and pendeo epitaxy is that GaN seeds on SiO_2_ in this work are epitaxially separated from a substrate while GaN seeds in pendeo epitaxy are epitaxially connected to a substrate^3^.

Another feature to note in Fig. 3(a) is that the surface of GaN is a little bit rough. In order to investigate the physical origin of this surface roughness, another regrown sample was KOH-etched. As Fig. 3(e) and (f) illustrate, the surface morphology before and after KOH etching reveals that the surface roughness is mainly due to the mixed polarity. Then, a question may arise: what is the physical origin of the mixed polarity. This seemingly puzzling situation is related to the finite etching speed of N-polar domains and etching nonuniformity across the sample. In other words, although a sample for the regrowth went through several-hour-long wet etching, it turned out that N-polar domains were not completely etched out on certain window regions. For example, there were still unetched N-polar domains after 5-hour wet etching as illustrated in Fig. 2(h). Thus, the regrown sample had both Ga- and N-polar seed domains in such a way that the surface got roughened.

Although the microstructural analysis revealed the validity of PILOSWER of GaN, the actual separation of a regrown film by HF-based chemical liftoff has been hindered supposedly because of some residual GaN domains on window regions, which connects GaN on SiO_2_ and the sapphire substrate. If these residual GaN domains on window regions are N-polar, further adjustment of experimental conditions would allow this film to be chemically lifted off. There are three evidences why these residual GaN domains on window regions are thought to be N-polar. First, the shape of the residual GaN domains on window regions is similar to a pyramidal hillock, which is the typical morphology when N-polar GaN surface is wet-etched. Second, if the residual GaN domains on window regions had been Ga-polar, the regrown sample should not have N-polarity at all, which is inconsistent with the mixed-polarity of the regrown sample. Third, as illustrated in Fig. 2(h), some N-polar domains still survived 5-hour-long-etching. The survival from several-hour-long wet etching does not necessarily imply the polarity of that domain to be Ga-polar.

**Figure 4:**
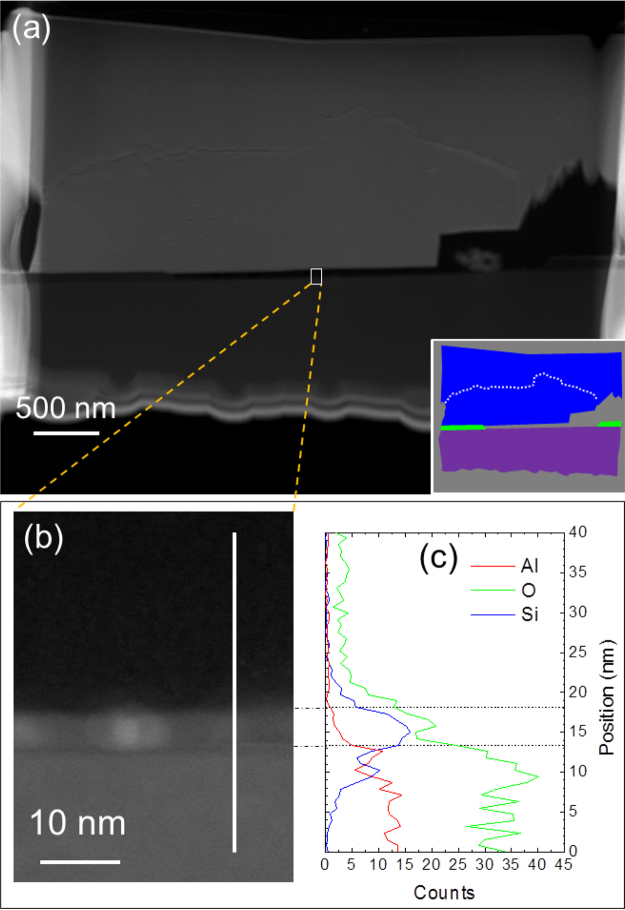
(**a**) A cross-sectional SEI of regrown GaN obtained by scanning transmission electron microscopy. An inset is a color-coded schematic diagram of the cross-sectional view. The regions in blue, green, violet represent GaN, SiO_2_, and sapphire. A dashed white line marks a crack caused during sample preparation. (**b**) A magnified image of the region marked by a white rectangle in (**a**). (**c**) An EDX line profile measured along a 40-nm-long white line marked in (**b**). A 4∼5-nm-thick layer on top of a sapphire surface consists of mainly Si and O with a small trace of Al.

For detailed analysis of sapphire surface passivation, energy dispersive X-ray spectroscopy (EDX) was measured. A regrown GaN sample was sliced and ion-milled for EDX measurement as illustrated in Fig. 4(a). A clear gap is seen between GaN and sapphire. A magnified image in Fig. 4(b) reveals that a 4$\sim$5-nm-thick layer exists on top of a sapphire surface. As shown Fig. 4(c), an EDX line profile measured along a white line in Fig. 4(b) reveals that silicon was oxidized on top of a sapphire surface. This oxide layer must have served as a mask layer during the regrowth and prohibited GaN from nucleating on a sapphire surface.

**Figure 5:**
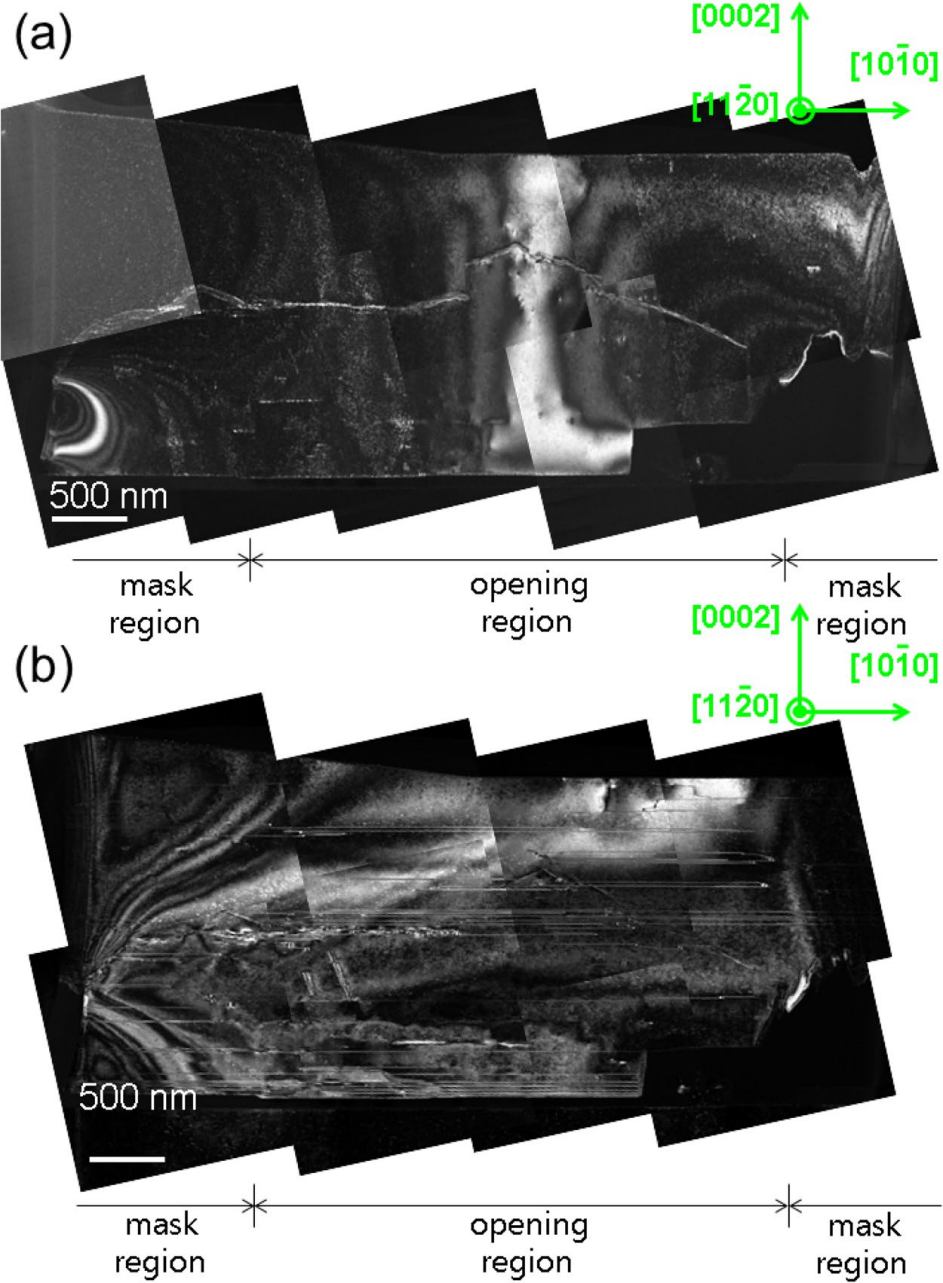
Cross-sectional TEM images of (**a**) g = 0002, (**b**) g = 10$\bar{{\rm{1}}}$0. Crystallographic orientations of GaN are labeled in green. No threading dislocations were observed in either mask or opening region. There were, however, some basal stacking faults.

To assess how effectively TDs can be eliminated in PILOSWER, TEM measurement was made with the sample shown in Fig. 4(a). Figure 5(a) reveals no sign of TDs in this film. On the other hand, there were some basal stacking faults as illustrated in Fig. 5(b). From the invisibility of stacking faults with g = 0002, these stacking faults are considered to be I_2_-type basal stacking faults^25^. Effective elimination of TDs in our experiment can be attributed to the fact that vertical sidewalls of GaN domains were formed during the first step (initial growth) and after the second step (selective etching), which is based on the following argument. Based on facet-controlled epitaxial lateral overgrowth (FACELO), two different distributions of TDs can be observed depending on the initially developed facet type when the stripe mask pattern is along the 〈1$\bar{{\rm{1}}}$00〉 direction of GaN: (1) TDs are mainly in mask regions when the initial domain facet is enclosed by {11$\bar{{\rm{2}}}$2} or (2) TDs are mainly in the window regions when the initial domain facet is enclosed by {0001} and vertical side facets^2,21–23^. In terms of overall density of TDs, the case (1) has much less TDs. However, if considered only in the mask region, the case (2) may be more effective in reduction of TDs. In the previous report on the selective area growth, Ga-polar facet was found to emerge only at high temperature and atmospheric pressure while N-polar facet was stable nearly most of experimental conditions^26^. Thus, the selective growth of N-polar GaN in window regions in this experiment, where flat top N-polar facet and vertical sidewalls were formed, corresponds to the case (2) of FACELO in such a way that TDs in N-polar GaN grown in window openings were not expected to bend toward the mask regions. Thus, selective wet etching of N-polar regions is thought to effectively remove most of TDs.

In conclusion, we proposed and demonstrated PILOSWER of GaN, which can be adopted for fabricating a GaN film, which is epitaxially separated from but physically attached to a substrate. In addition, it turned out that TDs were effectively removed during chemical wet etching. A GaN thin film grown in this manner is expected to be epitaxially decoupled from the sapphire substrate, so that it can serve as an excellent platform for fabricating devices readily separable from the substrate or a seed template for a thicker free-standing GaN substrate.

## Methods

100-nm-thick SiO_2_ was deposited on a *c*-plane sapphire substrate, and hexagonally arranged circular openings (2∼4 *μ*m diameter and 6∼8 *μ*m separation) were lithographically patterned. Growth of GaN was carried out by using hydride vapor phase epitaxy with N_2_ as a carrier gas. Each GaN sample was grown in two separate times: the growth of N-polar GaN on circular openings and polarity-inverted GaN on SiO_2_ near the circular boundary (initial growth) and subsequent growth after selective chemical wet etching (regrowth). During the initial growth, the growth was carried out at 900∼970 °C with 5∼10 sccm of HCl and 80∼4000 sccm of NH_3_ flow rates. In the regrowth, the growth temperature was at 1000 °C with 10 sccm of HCl and 200∼1000 sccm of NH_3_ flow rates.

Due to different chemical reactivity, it is well known that Ga- and N-polar surfaces have very different surface morphologies upon KOH or H_3_PO_4_ wet etching. Upon wet chemical etching, a Ga-polar surface remains to be flat, but a N-polar surface is filled with pyramidal hillocks^16,17,27,28^. In this work, wet chemical etching was used for polarity identification of GaN. Wet chemical etching of GaN was carried out with either 2 M KOH solution at 50 °C or 85 % H_3_PO_4_ solution at 85 °C.

For selective passivation, Si was sputtered for 4 minutes on a GaN/sapphire template with 30 W power and 1 × 10^−3^ torr in Ar gas. Then, this sample was etched in 2 M KOH solution at 50 °C for 15 minutes for partial removal of Si.

## Data Availability

All data generated or analysed during this study are included in this published article.
